# Natriuretic Effect of Dapagliflozin in Cirrhosis With Ascites: A Randomized, Placebo‐Controlled Crossover Trial

**DOI:** 10.1155/ijh/9503323

**Published:** 2026-04-08

**Authors:** Nattaporn Kongphakdee, Setthachai Piewchan, Kanokporn Sanpawithayakul, Soonthorn Chonprasertsuk, Sith Siramolpiwat

**Affiliations:** ^1^ Division of Gastroenterology, Department of Internal Medicine, Faculty of Medicine, Thammasat University, Pathumthani, Thailand, tu.ac.th; ^2^ Division of Gastroenterology, Department of Medicine, Faculty of Medicine, Naresuan University, Phitsanulok, Thailand, nu.ac.th; ^3^ Division of Endocrinology and Metabolism, Department of Internal Medicine, Faculty of Medicine, Thammasat University, Pathumthani, Thailand, tu.ac.th; ^4^ Department of Clinical Epidemiology, Faculty of Medicine, Thammasat University, Pathumthani, Thailand, tu.ac.th; ^5^ Chulabhorn International College of Medicine (CICM), Thammasat University, Pathumthani, Thailand, tu.ac.th

**Keywords:** ascites, cirrhosis, dapagliflozin, natriuresis, SGLT2 inhibitor

## Abstract

**Background:**

Ascites is a frequent complication of decompensated liver cirrhosis and is associated with a poor prognosis. Dapagliflozin, a sodium‐glucose cotransporter 2 (SGLT2) inhibitor, induces osmotic diuresis and natriuresis, with established use in heart failure. However, studies on SGLT2 inhibitors in cirrhotic patients with ascites are limited.

**Objectives:**

The objective of this study is to evaluate changes in urine parameters in cirrhotic patients with ascites treated with dapagliflozin.

**Methods:**

This randomized, double‐blind, placebo‐controlled, crossover trial enrolled 10 patients with Child–Pugh Class B or C cirrhosis who had persistently moderate or tense ascites. Participants received dapagliflozin or placebo for 4 weeks, followed by a 2‐week washout, and then crossed over to alternate treatment. The primary outcome was the change in 24‐h urine sodium (UNa) excretion on Days 0, 3, and 28. Secondary outcomes included changes in 24‐h urine volume (UV) and serum sodium.

**Results:**

Dapagliflozin significantly increased 24‐h urinary sodium excretion at Day 3 compared with placebo (treatment difference +35.1 mmol/day; 95% CI 17.5–52.7; *p* < 0.001), indicating a transient natriuretic effect. This effect was not sustained at Day 28 (*p* = 0.42). There were no significant changes in 24‐h UV or serum sodium at either time point. At the end of the dapagliflozin phase, 5 of 7 evaluable participants demonstrated improvement in ascites grade, whereas 2 did not. One serious adverse event occurred; one participant died during the washout period after completing the placebo phase due to variceal bleeding complicated by spontaneous bacterial peritonitis.

**Conclusions:**

In cirrhotic patients with persistent ascites, dapagliflozin was associated with a transient increase in 24‐h urinary sodium excretion, without significant sustained effects on urine volume or serum sodium. Dapagliflozin was generally well tolerated in this small pilot trial.

**Trial Registration:**

Thai Clinical Trial Registry: TCTR20241016007

## 1. Introduction

Ascites is the most common clinical complication of cirrhosis, accounting for approximately 50% of decompensating events [1, 2]. Ascites development is associated with a significant increase in morbidity, mortality, and poor quality of life [1, 3]. Conventional management strategies for cirrhotic ascites typically involve dietary sodium restriction, diuretic therapy, and therapeutic abdominal paracentesis. However, these treatments are often associated with limitations, including potential complications and the development of diuretic resistance, highlighting the need for novel therapeutic approaches [1, 4].

Dapagliflozin, a sodium‐glucose cotransporter 2 (SGLT2) inhibitor, exerts its pharmacological action by selectively inhibiting the reabsorption of glucose and sodium in the proximal convoluted tubule of the kidney [5]. This mechanism leads to increased urinary excretion of both water and sodium, resulting in osmotic diuresis and natriuresis. SGLT2 inhibitors have demonstrated significant therapeutic benefits in patients with heart failure by promoting fluid excretion [5]. Importantly, liver cirrhosis shares common pathophysiological pathways with heart failure, characterized by activation of the renin–angiotensin–aldosterone system (RAAS), sympathetic nervous system activation, and increased vasopressin secretion. These overlapping mechanisms suggest that SGLT2 inhibitors may offer a promising therapeutic avenue for patients with difficult‐to‐control ascites, irrespective of their diabetes status.

Despite the compelling theoretical rationale, studies investigating the use of SGLT2 inhibitors in liver cirrhosis with ascites have been primarily limited to case reports [6, 7]. Therefore, the present study is aimed at systematically investigating the effect of dapagliflozin on urine sodium (UNa) and urine volume (UV) excretion in patients with liver cirrhosis and ascites, including both diabetic and nondiabetic individuals. Specifically, we evaluated 24‐h UNa and 24‐h UV on Days 0, 3, and 28 to assess osmotic diuresis and natriuresis induced by dapagliflozin in this patient population.

## 2. Materials and Methods

### 2.1. Study Design

This was a randomized, double‐blind, placebo‐controlled, crossover trial conducted in patients with cirrhosis and ascites from August 2024 to March 2025 at the Department of Medicine, Thammasat University Hospital, Pathumthani, and Naresuan University Hospital, Phitsanulok, Thailand. The trial was approved by the Human Research Ethics Committee of Thammasat University (first posted on 16/10/2024). The study protocol conforms to the ethical guidelines of the 1975 Declaration of Helsinki.

### 2.2. Study Population

The inclusion criteria were as follows: (1) Child–Pugh Class B or C cirrhosis diagnosed by liver biopsy or a combination of clinical, biochemical, ultrasonographic, and endoscopic findings; (2) moderate or tense ascites defined as Grade 2 or 3 ascites according to previously established criteria [1]; (3) being on stable doses of diuretics for at least 1 month; and (4) written informed consent. The exclusion criteria were as follows: (1) chronic kidney disease (estimated glomerular filtration rate [eGFR] < 30 mL/min/1.73m^2^); (2) previous use of SGLT2 inhibitors within the past 12 months; (3) previous history of ketoacidosis or HbA1C > 9*%*; (4) active malignancy including hepatocellular carcinoma; (5) history of gastrointestinal bleeding, overt hepatic encephalopathy, or infection within 1 month; (6) require dosage change in medications that affect volume status (e.g., beta‐blockers and ACE inhibitors) within 1 month; (7) severe chronic heart or pulmonary diseases; and (8) anatomical urinary defect predisposing to urinary tract infection.

### 2.3. Study Protocol

#### 2.3.1. Randomization and Allocation

Eligible participants were screened based on predefined inclusion and exclusion criteria. Baseline data collection included a comprehensive medical history, physical examination, and standard laboratory investigations. Eligible participants were subsequently randomized in a 1:1 ratio into two parallel crossover sequences (Sequence A and Sequence B) using a computer‐generated randomization list. Randomization codes were generated by a research assistant who was not involved in the participant recruitment, clinical assessment, or outcome evaluation. Allocation assignments were concealed using sequentially numbered, opaque, sealed envelopes to maintain allocation concealment. An independent research assistant, who was also blinded to the study allocation sequence, was responsible for preparing and labeling the study interventions. Throughout the trial, all investigators, study coordinators, clinical assessors, and participants were blinded to the group allocation and the identity of the study drugs. Clinical follow‐up visits and data collection were conducted by blinded study researchers.

#### 2.3.2. Study Intervention

Throughout the study, participants continued to receive standard treatments for cirrhosis and ascites, including dietary sodium restriction and medical therapy. Briefly, a combination of furosemide and spironolactone was administered with dose titration in a 40:100 ratio, targeting a weight reduction of 0.5 kg/day in individuals without pedal edema and 1 kg/day in those with pedal edema. Large volume paracentesis with albumin infusion (8 g/L of ascites fluid removed) was performed for symptomatic Grade 3 ascites.

**Figure 1:**
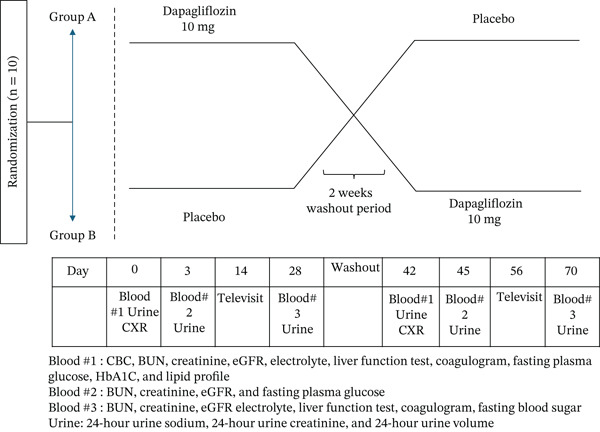
Flow diagram of the study.

In addition, all participants received two sequential blind interventions as part of a two‐period crossover design. The products included dapagliflozin 10 mg and matching placebo. The placebo was prepared by the Faculty of Pharmacy, Chiang Mai University, Thailand, and consisted primarily of inert flour manufactured to resemble dapagliflozin in appearance, size, and color. Each study drug was provided in a bottle containing 28 tablets, with instructions for oral administration of one tablet once daily before breakfast. Participants in Sequence A received dapagliflozin for 4 weeks, followed by a 2‐week washout period, and then crossed over to receive a placebo for 4 weeks. Conversely, Sequence B received a placebo first, followed by the same washout period and then dapagliflozin for 4 weeks. A telephone visit was conducted on Day 14 of each treatment phase to assess symptoms and monitor adverse events (Figure 1). The blinding of the study drugs was maintained throughout the study period.

The baseline assessment consisted of standard laboratory investigations, including 24‐h UNa, 24‐h urine creatinine (UCr), and 24‐h UV. Participants were subsequently followed up at outpatient clinics on Days 3, 14, and 28 during each study phase. To assess renal and natriuretic responses to the intervention, 24‐h urine collections were performed on Days 3 and 28 following the initiation of each treatment period.

#### 2.3.3. Study Outcomes

The primary outcome was the mean difference in 24‐h UNa between dapagliflozin and placebo on Days 3 and 28. We selected 24‐h UNa as the primary endpoint because impaired sodium excretion is central to ascites formation in cirrhosis, and UNa is routinely used in clinical practice to assess diuretic response and sodium balance. Given the short treatment periods in this crossover study, we considered UNa a sensitive mechanistic marker of changes in renal sodium handling. Secondary outcomes included the mean differences in 24‐h UV and serum sodium levels between dapagliflozin and placebo during the corresponding treatment periods.

The completeness of 24‐h urine collections was assessed using urinary creatinine excretion thresholds (< 15 mg/kg/day for women and < 20 mg/kg/day for men were considered incomplete). Primary analyses were conducted using evaluable urine collections. Sensitivity analyses including all available data are presented in Table S1.

#### 2.3.4. Statistical Analysis

Continuous variables were expressed as mean ± standard deviation (SD) or median with interquartile range (IQR), as appropriate. For crossover analyses, treatment effects were estimated using generalized estimating equations (GEE) to account for within‐subject correlation, with fixed effects for treatment and period. Sequence (treatment order) was explored in secondary analyses by inclusion as an additional fixed effect and was not associated with the primary outcome; therefore, sequence was not retained in the final model. Given the limited sample size of this pilot study, formal testing for carryover effects was not prespecified and was considered underpowered. Statistical significance was set at a *p* value of ≤ 0.05 for all analyses. All statistical analyses were performed using Stata Version 17.0.

To date, no studies have evaluated changes in UNa after the use of SGLT2 inhibitors in patients with cirrhosis and ascites; therefore, this investigation was designed as a pilot study. The sample size was determined according to a previously described method, which estimates the sample size for a pilot randomized trial to minimize the total number of participants required for both the pilot and subsequent main trial for a continuous outcome, based on the standardized difference from large‐scale studies [8]. An *α*‐error of 0.05 and a *β*‐error of 0.10 were applied, resulting in a target sample size of 10 participants in total. In the two‐sequence crossover design, this corresponded to five participants allocated to each treatment sequence. All randomized participants were included in the safety analysis. Efficacy analyses were performed using all available data according to treatment exposure.

## 3. Results

### 3.1. Baseline Characteristics

Ten participants were enrolled and randomized: five to Sequence A (dapagliflozin followed by placebo) and five to Sequence B (placebo followed by dapagliflozin). Baseline demographic and laboratory characteristics of the randomized cohort are presented in Table 1.

**Table 1:** Baseline characteristics and laboratory parameters of participants (*N* = 10).

Variables	*M* *e* *a* *n* ± *S* *D*
Male sex, *n* (%)	6 (60%)
Age (years)	66.4 ± 3.4
Weight (kg)	57.7 ± 10.7
DM, *n* (%)	5 (50%)

Etiology of cirrhosis, *n* (%)	
‐ Alcohol	4 (40%)
‐ Hepatitis C	3 (30%)
‐ Hepatitis B	2 (20%)
‐ MASLD	1 (10%)

Grade of ascites, 2/3, *n* (%)	8 (80%)/2 (20%)
Child–Pugh, B/C, *n* (%)	7 (70%)/3 (30%)
Child–Pugh score	8.9 ± 1.5
MELD score	12.1 ± 3.2
MELD‐Na score	14.9 ± 3.8

Laboratory parameters	
‐ Hb (g/dl)	9.4 ± 1.7
‐ Platelet (x10^3^/ul)	99.5 ± 40.8
‐ BUN (mg/dL)	16.6 ± 9.2
‐ Creatinine (mg/dL)	1.08 ± 0.4
‐ Sodium (mEq/L)	133.9 ± 3.7
‐ Potassium (mEq/L)	4.5 ± 0.5
‐ AST (U/L)	54.2 ± 20.1
‐ ALT (U/L)	27.4 ± 22.8
‐ Alkaline phosphatase (U/L)	128.1 ± 22.5
‐ Albumin (g/dL)	2.9 ± 0.4
‐ Total bilirubin (mg/dL)	1.9 ± 1.1
‐ Prothrombin time (sec)	14.2 ± 2.0
‐ FPG (mg/dL)	134.3 ± 32.8
‐ HbA1C (%)	5.7 ± 0.9

Diuretics use	
‐ Spironolactone dose (mg/day)	47.5 ± 62.9
‐ Furosemide dose (mg/day)	24.0 ± 26.3

Abbreviations: ALT, alanine transaminase; AST, aspartate aminotransferase; BUN, blood urea nitrogen; DM, diabetes mellitus; FPG, fasting plasma glucose; Hb, hemoglobin; MASLD, metabolic dysfunction‐associated steatotic liver disease; MELD, the Model for End‐Stage Liver Disease.

The mean age was 66.4 ± 3.4 years. The etiologies of cirrhosis included alcohol‐related (40%), hepatitis C (30%), hepatitis B (20%), and metabolic dysfunction‐associated steatotic liver disease (MASLD) (10%). Regarding disease severity, 70% and 30% were classified as Child–Pugh B and C, respectively. The mean Model for End‐stage Liver Disease (MELD) and MELD‐Na scores were 12.1 ± 3.2 and 14.9 ± 3.8, respectively.

The ascites grading was 2 and 3 in 8 (80%) and 2 (20%) patients, respectively. All patients received spironolactone during the study, whereas 4 (40%) received concomitant furosemide. The average daily doses of spironolactone and furosemide were 47.5 ± 62.9 and 24 ± 26.3 mg, respectively. Three participants (30%) had concurrent hepatic hydrothorax at enrollment. Five participants (50%) had diabetes mellitus and were treated with metformin (*n* = 3) or glipizide (*n* = 2). The mean fasting plasma glucose was 134.3 ± 32.8 mg/dL, and the mean HbA1c was 5.7*%* ± 0.9*%*. At baseline, the mean 24‐h urinary sodium excretion was 95.7 ± 34.1 mmol/day, the mean 24‐h UV was 1437.5 ± 513.9 mL, and the mean serum sodium level was 133.9 ± 3.7 mmol/L.

**Figure 2:**
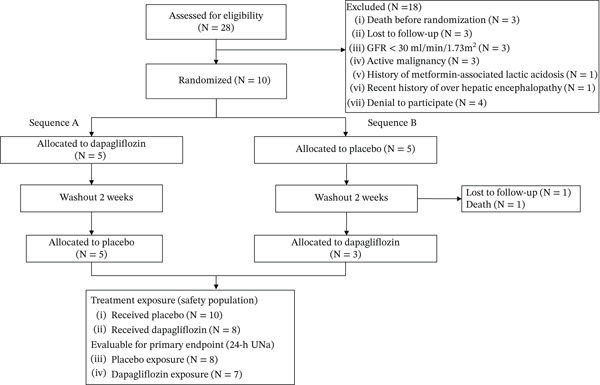
CONSORT diagram.

For the primary endpoint, evaluable 24‐h urinary sodium data were available from seven participants during dapagliflozin exposure and eight during placebo exposure. Six participants contributed complete paired data across both treatment periods and formed the primary within‐subject comparison for the crossover analysis. One participant died during the washout period after completing the placebo phase, one was lost to follow‐up before crossover, and one 24‐h urine collection was deemed incomplete (Figure 2, CONSORT diagram).

### 3.2. 24‐H Urine Collection

Compared with placebo, dapagliflozin induced a significant increase in 24‐h UNa on Day 3 (141.5 ± 66 vs. 110.4 ± 44 mmol/day; mean difference 35.10, 95% CI, 17.50–52.71; *p* < 0.001). However, this effect was not sustained on Day 28, with no significant difference observed between groups (112.9 ± 54.6 vs. 106.7 ± 52.9 mmol/day; mean difference 7.44, 95% CI, −10.66 to 25.55; *p* = 0.42) (Table 2). Regarding 24‐h UV, there was no significant difference in 24‐h UV between dapagliflozin and placebo at Day 3 or Day 28. (Day 3; 1682.9 ± 602.3 vs. 1587.5 ± 581.1 mL/day; mean difference 96.55, 95% CI, −187.92 to 381.03; *p* = 0.51, and Day 28; 1592.9 ± 680.3 vs. 1287.5 ± 484.6 mL/day; mean difference 302.53, 95% CI, −64.39 to 669.45; *p* = 0.11).

**Table 2:** Changes in 24‐h urine parameters.

Parameters	Dapagliflozin (*n* = 7) (SD/IQR)	Placebo (*n* = 8) (SD/IQR)	Treatment difference^a^ (95% CI)	p value
24‐h urine sodium, mmol/day				
Baseline	119.3 (40.7)	104.3 (57.1)		
Day 3	141.5 (66)	110.4 (44)	35.10 (17.50 to 52.71)	< 0.001
Day 28	112.9 (54.6)	106.7 (52.9)	7.44 (−10.66 to 25.55)	0.42

24‐h urine creatinine, mg/day				
Baseline	703.7 (292.6)	831.4 (350.4)		
Day 3	820.7 (357.8)	832.1 (362.3)	−46.32 (−182.77 to 90.11)	0.51
Day 28	870.6 (330.8)	823.0 (407.8)	21.1 (−88.57 to 130.76)	0.71

Fractional excretion of sodium,%^b^				
Baseline	0.11 (0.08, 0.17)	0.09 (0.08, 0.13)		
Day 3	0.12 (0.07, 0.23)	0.1 (0.07, 0.18)	0.07 (−0.01 to 0.14)	0.09
Day 28	0.09 (0.08, 0.21)	0.11 (0.08, 0.17)	−0.01 (−0.03 to 0.00)	0.09

24‐h urine volume, mL				
Baseline	1507.1 (437.3)	1462.5 (651.2)		
Day 3	1682.9 (602.3)	1587.5 (581.1)	96.55 (−187.92 to 381.03)	0.51
Day 28	1592.9 (680.3)	1287.5 (484.6)	302.53 (−64.39 to 669.45)	0.11

^a^Treatment effect estimates represent model‐based differences (dapagliflozin—placebo) derived from generalized estimating equations (GEE) including treatment and period effects to account for within‐subject correlation in the crossover design. Consequently, estimates may not equal simple arithmetic differences of the summarized values.

^b^Values are presented as median (IQR) for nonnormally distributed variables. Treatment effects were estimated using GEE on the original measurement scale.

The fractional excretion of sodium (FeNa) did not differ significantly between dapagliflozin and placebo at either time point. Model‐based treatment differences were 0.07 (95% CI −0.01 to 0.14; *p* = 0.09) on Day 3 and −0.01 (95% CI −0.03 to 0.00; *p* = 0.09) on Day 28 (Table 2).

### 3.3. Serum Sodium Levels and Other Laboratory Parameters

Table 3 shows changes in clinical and laboratory parameters between the two groups. Dapagliflozin did not significantly change serum sodium concentrations compared with placebo at either time point. On Day 3, serum sodium was 135 ± 3.8 mmol/L in the dapagliflozin group versus 134.6 ± 4.2 mmol/L in the placebo group (mean difference −0.57, 95% CI, −2.2 to 1.1; *p* = 0.49). On Day 28, levels were 135.4 ± 2.8 and 134.3 ± 5.5 mmol/L, respectively (mean difference −1.51, 95% CI, −4.2 to 1.1; *p* = 0.26).

**Table 3:** Changes in clinical and laboratory parameters.

	Dapagliflozin (*n* = 7) (SD/IQR)	Placebo (*n* = 8) (SD/IQR)	Treatment difference^a^ (95% CI)	p value
Body weight, kg				
Baseline	59.8 (12.9)	58.3 (14.2)		
Day 3	59.2 (12.6)	57.8 (12.7)	0.34 (−2.21 to 2.89)	0.80
Day 28	60.1 (15.2)	58.4 (12.5)	0.32 (−2.20 to 2.85)	0.80
Child–Pugh score				
Baseline	8.0 (1.4)	8.4 (2.0)		
Day 28	7.3 (1.4)	7.5 (1.8)	−0.09 (−0.47 to 0.29)	0.65
MELD				
Baseline	11.7 (2.9)	12.3 (3.2)		
Day 28	11.6 (4.0)	13.1 (2.6)	−1.28 (−2.72 to 0.16)	0.08
MELD‐Na				
Baseline	15.3 (4.1)	15.5 (3.8)		
Day 28	14.6 (4.1)	15.9 (4.1)	−1.92 (−2.30 to −1.53)	<0.001
eGFR, mL/min/1.73m^2^				
Baseline	70.2 (26.7)	65.6 (24.1)		
Day 3	69.4 (25.7)	66.3 (24.1)	2.83 (−3.91 to 9.56)	0.41
Day 28	68.2 (26.5)	63.2 (22.7)	5.19 (−4.76 to 15.14)	0.31
Serum creatinine, mg/dL				
Baseline	1.06 (0.39)	1.11 (0.33)		
Day 3	1.10 (0.41)	1.10 (0.32)	−0.02 (−0.13 to 0.09)	0.74
Day 28	1.13 (0.40)	1.14 (0.36)	−0.03 (−0.20 to 0.14)	0.72
Serum sodium, mmol/L				
Baseline	134.3 (3.8)	134.8 (2.7)		
Day 3	135.0 (3.8)	134.6 (4.2)	0.57 (−1.06 to 2.20)	0.49
Day 28	135.4 (2.8)	134.3 (5.5)	1.51 (−1.12 to 4.15)	0.26
Serum potassium, mmol/L				
Baseline	4.4 (0.5)	4.5 (0.4)		
Day 3	4.3 (0.7)	4.4 (0.4)	−0.03 (−0.39 to 0.32)	0.86
Day 28	4.6 (0.5)	4.5 (0.4)	0.03 (−0.51 to 0.58)	0.90
FPG, mg/dL				
Baseline	114.9 (33.1)	121.4 (33.1)		
Day 3	118.6 (52.3)	107.7 (21.4)	10.71 (−30.50 to 51.93)	0.61
Day 28	111.0 (18.8)	109.8 (24.9)	0.83 (−9.12 to 10.78)	0.87

^a^Treatment difference represents dapagliflozin—placebo and was estimated using generalized estimating equations including treatment and period effects to account for within‐subject correlation in the crossover design.

Abbreviations: eGFR, estimated glomerular filtration rate; FPG, fasting plasma glucose; MELD, the Model for End‐Stage Liver Disease.

No significant differences were observed in FPG between groups on Day 3 (118.6 ± 52.3 vs. 107.7 ± 21.4 mg/dL; mean difference −10.71, 95% CI, −51.9 to 30.5; *p* = 0.61) or Day 28 (111 ± 18.8 vs. 109.8 ± 24.9 mg/dL; mean difference −0.83, 95% CI, −10.8 to 9.1; *p* = 0.87). No hypoglycemia occurred during the study period. Additionally, serum creatinine, eGFR, and serum potassium levels remained stable at both time points, with no statistically significant differences between groups.

### 3.4. Clinical Outcomes

There was no significant difference in body weight on day 3 (59.2 ± 12.6 vs. 57.8 ± 12.7 kg; *p* = 0.8) or Day 28 (60.1 ± 15.2 vs. 58.4 ± 12.5 kg; *p* = 0.8). Regarding the cirrhotic severity, there was no significant difference in the Child–Pugh and MELD scores on Day 28; however, the MELD‐Na score on Day 28 was significantly lower in the dapagliflozin group (14.6 ± 4.1 vs. 15.9 ± 4.1; mean difference −1.92, 95% CI, −2.30 to −1.53; *p* < 0.001).

At the end of the dapagliflozin treatment period, 5 of 7 evaluable participants demonstrated an improvement in ascites grade, whereas 2 did not; one of these required large‐volume paracenteses with albumin administration during follow‐up.

Regarding hepatic hydrothorax, complete resolution was observed in one participant during dapagliflozin exposure, accompanied by an increase in 24‐h urinary sodium excretion from 93.2 to 236.5 mmol/day by Day 3. During placebo exposure, one participant achieved complete resolution, whereas another demonstrated partial improvement that progressed to complete resolution 70 days after completion of the 4‐week dapagliflozin period.

### 3.5. Adverse Events

During the study period, no participants experienced urinary tract infection, hypotension, ketoacidosis, or bone fractures. One serious adverse event occurred: A participant died during the washout phase after completing the placebo treatment period due to variceal bleeding complicated by spontaneous bacterial peritonitis. This participant did not receive dapagliflozin.

## 4. Discussion

This randomized, double‐blind, placebo‐controlled, crossover trial is aimed at investigating the short‐term effects of dapagliflozin on ascites in cirrhotic patients. The main finding was a significant increase in 24‐h UNa excretion by Day 3 following dapagliflozin administration. However, this initial natriuretic effect was not sustained, as no significant changes in 24‐h UNa, FeNa, and UV were detected by Day 28. These results suggest a transient early natriuretic response to dapagliflozin in this patient population.

Traditional methods for assessing ascites treatment response typically include clinical evaluation, imaging studies, and 24‐h UNa measurement. Given the brief 4‐week duration of each study phase in our trial, the clinical and radiological assessments were likely insufficiently sensitive to capture subtle changes in ascites. Consequently, 24‐hour UNa was selected as a surrogate marker to evaluate early improvement in ascites. This approach is supported by a recent study that demonstrated an association between 24‐h UNa level and the grading of ascites [9].

The transient increase in 24‐h UNa observed at Day 3, followed by attenuation by Day 28, is consistent with prior studies of SGLT2 inhibitors in both diabetic and heart failure populations [10–13]. Early natriuresis likely reflects inhibition of proximal tubular sodium–glucose cotransport, resulting in increased sodium delivery to the distal nephron. However, compensatory activation of distal sodium reabsorption mechanisms and neurohormonal pathways, particularly the renin–angiotensin–aldosterone system, may attenuate this effect over time [14, 15]. This adaptive response establishes a new equilibrium in sodium and volume homeostasis, thereby limiting sustained natriuresis. Our findings, including the absence of a significant increase in UNa and FeNa at Day 28, are therefore physiologically consistent with the known renal adaptation to SGLT2 inhibition.

Concomitant loop diuretic therapy may enhance the diuretic response to SGLT2 inhibitors. Prior studies have demonstrated increased UV and natriuresis when SGLT2 inhibitors were combined with loop diuretics, suggesting potential synergistic effects at different nephron segments. [16, 17] In our cohort, a sustained increase in 24‐h UV was not observed, which may partly reflect that only half of the patients were receiving loop diuretics, potentially limiting such synergistic effects.

Our findings support those of previous studies investigating the effects of SGLT2 inhibitors on serum sodium levels in decompensated cirrhotic patients. These studies showed an increase in serum sodium levels in hyponatremic patients, with no cases of hypernatremia observed in those with normal baseline sodium levels [6, 7, 18, 19]. Proposed mechanisms for this effect include osmotic diuresis, which promotes the excretion of electrolyte‐free water; a decrease in atrial natriuretic peptide (ANP) levels, thereby reducing sodium excretion; and increased arginine vasopressin (AVP) stimulation, facilitating water reabsorption to prevent dehydration and hypernatremia [20]. In our study, no participant experienced clinically significant fluctuations in serum sodium levels (neither hyponatremia nor hypernatremia) after receiving dapagliflozin.

Beyond the observed changes in urinary sodium excretion, our study also noted improvements in fluid retention, including ascites and hepatic hydrothorax. These findings are consistent with prior case series reporting improved ascites control and peripheral edema with SGLT2 inhibitors as add‐on therapy in cirrhosis [6, 7]. In addition, a recent prospective pilot study demonstrated that 6‐month dapagliflozin treatment in patients with recurrent ascites was associated with improved ascites control and reduced need for large‐volume paracentesis [21]. However, unlike prior studies that primarily evaluated longer‐term clinical outcomes, our crossover design allowed within‐patient assessment of short‐term renal sodium dynamics. The early increase in natriuresis followed by attenuation observed in our cohort provides mechanistic insight into renal adaptive responses in advanced cirrhosis and may help explain variability in sustained clinical benefit across studies.

In addition to ascites and hydrothorax, SGLT2 inhibitors show promise in various aspects of cirrhosis management. Recent studies suggest that SGLT2 inhibitors offer therapeutic benefits in chronic liver diseases through multiple mechanisms beyond their traditional diuretic effects, such as hepatoprotective effects through ketogenesis [22], sympathetic nervous system modulation [23], counteracting RAAS overactivation [24], and an antifibrotic agent [25]. Interestingly, a recent systematic review in patients with DM and MASLD found that SGLT2 inhibitors significantly reduced liver stiffness compared with other hypoglycemic agents. This improvement was more pronounced in patients with longer treatment durations and more severe baseline fibrosis [26]. Moreover, a recent randomized placebo‐controlled study has elucidated the beneficial effect of dapagliflozin treatment on histological outcomes in patients with metabolic dysfunction‐associated steatohepatitis (MASH) [27].

The safety profile of SGLT2 inhibitors in liver impairment has been previously described [28]. Based on pharmacokinetic properties, SGLT2 inhibitors undergo hepatic metabolism mainly through glucuronidation; therefore, patients with severe hepatic impairment could have an increase in drug exposure. Nevertheless, these medications are considerably safe and well tolerated in cirrhotic patients [20, 29]. Individual benefit‐risk assessment and dose titration are recommended in patients with severe liver impairment [28]. In our study, no patients experienced any adverse event related to SGLT2 inhibitors. However, in a recent trial, higher incidences of infection and acute kidney injury (mostly precipitated by sepsis) were reported in the dapagliflozin arm [21]. These findings underscore the importance of careful patient selection and close monitoring when administering SGLT2 inhibitors to cirrhotic patients.

Despite these promising findings, several limitations should be acknowledged. First, the small sample size limits statistical power and generalizability. This study was designed as a pilot mechanistic crossover trial to explore short‐term renal sodium dynamics in cirrhosis and ascites. Second, although all 10 participants were randomized, incomplete 24‐h urine collections and loss to follow‐up reduced the number of evaluable observations for the primary endpoint. To address this, predefined criteria were used to assess urine completeness, and sensitivity analyses including all available data yielded consistent results. Third, the short treatment duration precluded assessment of long‐term clinical outcomes, including sustained ascites control and long‐term safety. Fourth, advanced cirrhosis in some participants may have affected the reliability of 24‐h urine collections, and urine osmolality or free water clearance were not directly measured, limiting detailed assessment of water handling. Fifth, changes in composite scores such as MELD‐Na should be interpreted cautiously in small mechanistic crossover studies and were not primary outcomes of this trial. Finally, although a 2‐week washout period was incorporated; residual carryover effects cannot be entirely excluded in patients with altered drug metabolism. To mitigate this concern, crossover analyses included both treatment and period effects; however, the small sample size may have limited the power to detect subtle carryover effects. Despite these limitations, dapagliflozin appeared to have an acceptable short‐term safety profile in patients with ascites, with no significant adverse events observed. Future studies should address these limitations by enrolling a larger sample size, extending follow‐up periods, and incorporating additional clinical and laboratory parameters to comprehensively evaluate the effects of SGLT2 inhibitors on ascites and the overall outcomes in patients with chronic liver disease.

In conclusion, dapagliflozin demonstrated a short‐term improvement in urinary sodium excretion in cirrhotic patients with ascites. However, this effect appeared to be transient, with limited long‐term efficacy on UV and serum sodium levels, likely due to compensatory renal adaptations. Despite these modest outcomes, dapagliflozin appeared to have an acceptable short‐term safety profile, suggesting its potential as a supportive therapeutic option, particularly in combination with loop diuretics. Further research is warranted to elucidate its long‐term benefits, optimal use, and broader role in the comprehensive management of ascites and other complications in patients with cirrhosis.

**Nomenclature**
SGLT2: sodium‐glucose cotransporter 2
RAAS: renin‐angiotensin‐aldosterone system
UNa: urine sodium
UV: urine volume
UCr: urine creatinine
SD: standard deviation
MASLD: metabolic dysfunction‐associated steatotic liver disease
MELD: Model for End‐stage Liver Disease
DM: diabetes mellitus
FPG: fasting plasma glucose
FeNa: fractional excretion of sodium
NHE3: sodium‐hydrogen exchanger isoform 3
ANP: atrial natriuretic peptide
AVP: arginine vasopressin

## Author Contributions

N.K.: data curation, methodology, formal analysis, and writing the original draft. S.P.: data curation and editing the final draft. K.S.: methodology, formal analysis, project supervision, and editing the final draft. S.C.: supervision and editing the final draft. S.S.: conceptualization, methodology, formal analysis, funding acquisition, project supervision, and editing the final draft.

## Funding

This study was supported by the Gastrointestinal Association of Thailand (GAT) and AstraZeneca Pharmaceuticals Co. Ltd., Thailand.

## Ethics Statement

This study was conducted in accordance with the 1975 Helsinki Declaration and approved by the Institutional Review Board of Thammasat University. All participants provided written informed consent before participating in the study.

## Conflicts of Interest

The authors declare no conflicts of interest.

## Supporting information


**Supporting Information** Additional supporting information can be found online in the Supporting Information section. Table S1: Sensitivity analysis including all randomized participants and all 24‐h urine collections, analyzed using the prespecified crossover GEE model.

## Data Availability

The data that support the findings of this study are available from the corresponding author upon reasonable request.
