# Supplementary material for: Natriuretic Effect of Dapagliflozin in Cirrhosis With Ascites: A Randomized, Placebo‐Controlled Crossover Trial
Source: Int J Hepatol. 2026 Apr 8;2026:9503323. doi: 10.1155/ijh/9503323 (PMC13058925; doi:10.1155/ijh/9503323)
Supplement: Supplementary file 1 — Supporting Information Additional supporting information can be found online in the Supporting Information section. Table S1: Sensitivity analysis including all randomized participants and all 24‐h urine collections, analyzed using the prespecified crossover GEE model. [file IJH-2026-9503323-s001.docx]

**Supplementary files**

**Supplementary table 1:** Sensitivity analysis including all randomized participants and all 24-hour urine collections, analyzed using the prespecified crossover GEE model

| Parameters | Dapagliflozin (n=8)  (SD/IQR) | Placebo (n=10)  (SD/IQR) | Treatment Difference^*^ (95%CI) | p-value |
| --- | --- | --- | --- | --- |
| 24-h urine sodium, mmol/day  Baseline  Day 3  Day 28 | 108.2 (49.1)  125.8 (75.5)  99.1 (63.9) | 83.9 (66.1)  89.0 (59.6)  86.0 (63.8) | 35.57 (20.54 to 50.61)  9.49 (-6.11to 25.09) | <0.001  0.23 |
| 24-h urine creatinine, mg/day  Baseline  Day 3  Day 28 | 680.4 (278.8)  803.6 (334.8)  833.8 (323.5) | 769.6 (373.5)  803.6 (294.6)  787.4 (374.3) | -33.81 (-161.53 to 93.32)  39.89 (-61.88 to 141.67) | 0.60  0.44 |
| Fractional excretion of sodium,%^* *^  Baseline  Day 3  Day 28 | 0.10 (0.08, 0.16)  0.11 (0.05, 0.20)  0.09 (0.04, 0.15) | 0.08 (0.02, 0.11)  0.07 (0.05, 0.16)  0.09 (0.02, 0.13) | 0.07 (0.01 to 0.14)  0 (-0.03 to 0.03) | 0.06  0.97 |
| 24-h urine volume, mL  Baseline  Day 3  Day 28 | 1350 (1050, 1750)  1600 (1050, 2050)  1650 (700, 2025) | 1125 (500, 1950)  1350 (800, 1750)  1100 (600, 1700) | 151.07 (-132.25 to 433.40)  275.30 (-84.96 to 635.57) | 0.29  0.13 |
| Body weight, kg  Baseline  Day 3  Day 28 | 60.0 (12.0)  59.5 (11.7)  60.1 (14.1) | 58.2 (12.5)  57.9 (11.2)  58.2 (11.1) | 2.10 (-1.58 to 5.78)  2.13 (-2.51 to 6.78) | 0.26  0.64 |
| eGFR, mL/min/1.73m^2^  Baseline  Day 3  Day 28 | 69.2 (24.9)  64.6 (27.4)  62.6 (29.3) | 60.2 (24.3)  62.7 (22.9)  57.7 (23.6) | 1.69 (-6.17 to 9.54)  4.05 (-5.61 to 13.72) | 0.67  0.41 |
| Serum creatinine, mg/dL  Baseline  Day 3  Day 28 | 1.08 (0.37)  1.23 (0.52)  1.33 (0.67) | 1.25 (0.43)  1.18 (0.34)  1.33 (0.55) | 0.03 (-0.10 to 0.15)  0.01 (-0.17 to 0.18) | 0.70  0.95 |
| Serum sodium, mmol/L  Baseline  Day 3  Day 28 | 133.9 (3.7)  134.3 (4.1)  134.5 (3.7) | 133.1 (4.3)  133.1 (4.9)  133.3 (5.4) | 0.84 (-0.65 to 2.33)  1.58 (-0.97 to 4.12) | 0.27  0.23 |
| Serum potassium, mmol/L  Baseline  Day 3  Day 28 | 4.5 (0.5)  4.4 (0.7)  4.6 (0.5) | 4.6 (0.5)  4.5 (0.4)  4.5 (0.4) | 0.00 (-0.33 to 0.32)  0.05 (-0.43 to 0.54) | 0.98  0.83 |
| Fasting plasma glucose, mg/dL  Baseline  Day 3  Day 28 | 134.3 (32.8)  127.1 (54.0)  122.6 (37.2) | 123.0 (35.3)  121.7 (56.6)  115.7 (40.7) | 2.71 (-35.06 to 40.49)  1.16 (-7.53 to 9.84) | 0.89  0.79 |

*****Treatment difference represents Dapagliflozin − Placebo and was estimated using generalized estimating equations including treatment and period effects to account for within-subject correlation in the crossover design.

** Values are reported in as median (IQR) due to non-normal distribution.
